# Dermatomyositis: A Narrative Review of Skin as a Window to Muscle and Malignancy

**DOI:** 10.7759/cureus.88601

**Published:** 2025-07-23

**Authors:** Maria Cristofori, José C González-Rodríguez, Emmanuel E Cortés - Marín, Adipp Sallón, Jairo Sandoval

**Affiliations:** 1 General Practice, Universidad de Ciencias Médicas (UCIMED), San Jose, CRI; 2 Internal Medicine, Universidad de Costa Rica, San José, CRI; 3 Emergency Medicine, Hospital México, San Jose, CRI; 4 General Practice, Hospital México, San Jose, CRI

**Keywords:** autoimmunity, dermatomyositis, inflammatory myopathies, interstitial lung disease, myositis-specific antibodies, paraneoplastic syndrome

## Abstract

Dermatomyositis (DM) is a complex, systemic autoimmune disease characterized by hallmark cutaneous manifestations and inflammatory myopathy. It is associated with significant morbidity due to multisystem organ involvement and a well-established risk of underlying malignancy. This review provides a comprehensive update on the integrated pathophysiology, clinical subtypes, diagnostic strategies, and current management of DM, with a special emphasis on the modern, serology-driven approach to patient care. The pathogenesis is understood as an immune-mediated microangiopathy, with a central role for the type I interferon signaling pathway in driving tissue damage in both skin and muscle. The classification of DM has evolved beyond clinical presentation to a clinico-serological model where myositis-specific autoantibodies define distinct phenotypes with critical prognostic implications. For instance, anti-TIF1γ and anti-NXP2 antibodies are strongly associated with a high risk of malignancy, while anti-MDA5 antibodies identify patients at high risk for rapidly progressive interstitial lung disease. The diagnostic approach integrates clinical findings with serological profiling, imaging studies such as muscle MRI, and histopathology, while a risk-stratified cancer screening protocol is a mandatory component of initial management in adults. Treatment has advanced from a primary reliance on corticosteroids and conventional immunosuppressants to include targeted therapies, such as rituximab and, more recently, Janus kinase inhibitors, which have shown efficacy in treating refractory disease. Ultimately, a personalized and multidisciplinary approach, guided by the patient's specific autoantibody profile, is essential for optimizing long-term outcomes in this heterogeneous disease.

## Introduction and background

Dermatomyositis (DM) is an inflammatory myopathy characterized by the combination of a subacute onset of proximal muscle weakness accompanied by distinctive cutaneous lesions [1]. Far from being a benign condition, DM is linked to considerable morbidity, including progressive muscle disability and disabling skin lesions, and increased mortality rates, primarily driven by associated interstitial lung disease (ILD) and malignancy [1,2]. Its diagnosis requires a high degree of clinical suspicion due to the variability in its cutaneous, muscular, and extramuscular manifestations, which are now better understood through novel immunopathological classifications [3].

From a dermatological perspective, the cutaneous symptoms may precede, accompany, or occur independently of muscle involvement, as seen in amyopathic DM [2,4]. Classic lesions, such as Gottron's papules, the heliotrope sign, and poikiloderma in photo-exposed regions, constitute key findings with diagnostic value [1,2]. Beyond their clinical significance, these lesions indicate underlying immunopathological mechanisms, such as complement activation and microvascular damage, which are also present in other affected organs [5,6].

In this broader context, the strong paraneoplastic association of DM remains a primary concern, with up to 40% of adult patients developing an associated cancer [7]. Recent advances have transformed this statistical link into a clinically actionable, biomarker-driven field. The identification of autoantibodies like anti-TIF1-γ now serves as a highly specific marker for cancer-associated DM, mandating immediate and targeted oncological screening [8-10]. This paradigm shift, from generalized suspicion to precise, antibody-guided risk stratification, highlights the urgent need for clinicians to integrate these novelties into their diagnostic workflow to facilitate the early detection of hidden tumors [3].

The rapid evolution in the understanding of DM, from new pathophysiological insights to the clinical implementation of phenotype-specific biomarkers and targeted therapies, presents a significant challenge for clinicians trying to maintain an updated and integrated approach. This landscape raises a critical question: How can these multifaceted, rapidly evolving developments be synthesized into a coherent strategy for modern clinical practice? Answering this question, this review provides a comprehensive synthesis of these practice-changing developments. We will focus on how the new immunological classifications directly impact the diagnosis, prognosis, oncological screening, and treatment strategies for DM, equipping clinicians with the updated knowledge necessary to improve patient outcomes in this complex disease.

## Review

Methods

This article presents a narrative review based on a comprehensive literature search conducted as of June 2025. A non-systematic search was performed on major scientific databases, including PubMed/MEDLINE, Google Scholar, and SpringerLink. The search strategy employed a combination of keywords, including "Dermatomyositis," "inflammatory myopathies," "myositis-specific antibodies," "paraneoplastic syndrome," "interstitial lung disease," and "JAK inhibitors." Priority was given to recent high-impact publications, including international guidelines, systematic reviews, meta-analyses, and key original research. Foundational articles were also included to provide historical context. The selection of literature was based on its relevance to the topics of modern pathophysiology, clinico-serological classification, oncological screening, and emerging therapies, as judged by the authors' expertise in the field.

Integrated immunopathophysiology

DM is a complex autoimmune disease that necessitates an integrated approach, incorporating immunopathology, multisystem clinical manifestations, and recent advances in therapy. While its characteristic cutaneous lesions facilitate early clinical recognition, the systemic aspects of the disease, particularly muscle and lung involvement, along with its association with malignancy, introduce significant diagnostic and prognostic challenges [1]. The subsequent sections will elaborate on the primary pathophysiological, clinical, and immunological features of dermatomyositis, with a particular emphasis on its phenotypic heterogeneity, the role of autoantibodies as diagnostic tools, and contemporary therapeutic strategies.

Immunological Activation in Skin and Muscle

While historically understood primarily through its inflammatory presentation in skin and muscle, the modern conception of DM pathophysiology has been reshaped by the identification of the type I interferon pathway as a central, unifying mechanism [11]. DM is now recognized as a systemic autoimmune disease whose pathophysiology involves the coordinated activation of the innate and adaptive immune systems. Although the exact mechanisms have not been fully elucidated, current evidence suggests that the pathological process is initiated in genetically predisposed individuals following environmental triggers (such as viruses, drugs, or neoplasms), leading to an aberrant immune response mediated by T and B lymphocytes, type I interferons, and complement activation [11,12].

In muscle tissue, one of the characteristic findings is the deposition of the membrane attack complex (C5b-9) in endomysial capillaries, which reflects an immune-mediated microangiopathy [13]. This vascular damage leads to muscle hypoperfusion, fiber necrosis, and regeneration, which clinically manifests as symmetric and subacute muscle weakness. Simultaneously, a perivascular and perifascicular infiltration of CD4+ T cells and plasmacytoid dendritic cells has been documented, as well as the overexpression of major histocompatibility complex class I (MHC-I) molecules on non-necrotic muscle fibers [14].

In the skin, similar immunopathological mechanisms are observed. Classic cutaneous lesions, such as Gottron's papules and the heliotrope sign, also show deposits of C5b-9 and immunoglobulins along the dermoepidermal junction [15]. Apoptosis of keratinocytes in the basal layer of the epidermis, induced by type I interferons and mediated by cytotoxic T cells, is considered a key mechanism of epithelial damage [13]. Furthermore, the activation of dendritic cells and the local production of proinflammatory cytokines contribute to the perpetuation of cutaneous inflammation [16].

These pathogenic processes reflect an immunological convergence between skin and muscle, supported by a common transcriptomic signature dominated by the activation of type I interferon, which has been associated with greater clinical activity, tissue damage, and response to treatment. This molecular and cellular integration justifies considering dermatomyositis as a systemic inflammatory microvascular entity rather than the sum of independent organ manifestations [13,16].

Specific Autoantibodies and Paraneoplastic Mechanisms

Perhaps no area has seen more dramatic evolution in the last decade than the characterization of autoantibodies in DM. Myositis-specific autoantibodies (MSAs) and myositis-associated autoantibodies (MAAs) have revolutionized the clinical approach, shifting the field from a 'one-size-fits-all' diagnostic model to a highly stratified and personalized practice, enabling a more precise classification of clinical subtypes and facilitating the prediction of complications, such as ILD or occult malignancy. Although some have diagnostic value, many of these autoantibodies also possess key pathophysiological and prognostic implications [8].

Among the most studied MSAs, the anti-TIF1γ antibody is notable for its strong association with cancer in adults with dermatomyositis. Recent studies have confirmed a malignancy prevalence of up to 70% in this population, with a critical window during the first year following the DM diagnosis. Clinically, it is associated with severe cutaneous lesions, extensive poikiloderma, and scant pulmonary involvement [17-19].

Anti-NXP2, initially described in juvenile forms, has also been linked to a significant oncologic risk in adults, particularly in older men. This antibody is associated with extensive calcinosis and marked muscle weakness [20]. On the other hand, anti-Mi-2, although historically linked to the “classic” form of DM, is currently associated with a low risk of cancer and a better overall prognosis [21,22].

The anti-MDA5 antibody is found mainly in patients with amyopathic or hypomyopathic DM, with a high risk of rapidly progressive ILD and a poor respiratory prognosis, but with a generally low or non-significant oncologic risk [23]. In contrast, the less frequent anti-SAE1 has been identified in patients with a florid cutaneous onset and delayed muscle weakness. An increased cancer risk of up to 5 times that of the general population has been documented [24].

Within the group of MAAs, anti-Ro52 has been established as a marker of increased mortality in patients with ILD, especially when it coexists with other MSAs, such as anti-MDA5. This antibody is not specific to DM, but its presence predicts severe forms of pulmonary involvement, regardless of the clinical subtype [25]. Other MAAs, such as anti-PM/Scl and anti-Ku antibodies, usually appear in the context of overlap syndromes with systemic sclerosis or systemic lupus erythematosus. Although they are rarely associated with cancer, their recognition helps define therapeutic strategies and long-term follow-up [26].

Finally, antisynthetase antibodies (anti-Jo-1, PL-7, PL-12, among others), which are characteristic of antisynthetase syndrome, can coexist with DM-like cutaneous phenotypes. Their presence suggests an elevated risk of ILD, non-erosive arthritis, Raynaud's phenomenon, and mechanic's hands. Although their association with cancer is less clear, some subtypes, such as anti-PL-7, have been linked to more fibrosing and aggressive pulmonary disease [27].

From an immunological perspective, the autoantibodies implicated in DM, especially those targeting nuclear proteins (such as TIF1γ or NXP2), could participate directly in pathological processes through the recognition of tumor neoantigens, molecular mimicry, or the disruption of immunological tolerance. These mechanisms would partly explain the relationship between DM and neoplasia [18,20].

Clinical presentation and organ involvement

The complex interplay of microangiopathy, complement activation, and the interferon-mediated immune response, as discussed in the previous section, clinically manifests as a broad and heterogeneous spectrum of signs and symptoms. While skin and skeletal muscle involvement constitutes the diagnostic axis of dermatomyositis, the disease can be multisystemic, affecting the lungs, cardiovascular system, and gastrointestinal tract [1,13]. The precise recognition of these manifestations is crucial not only for diagnosis but also for prognostic stratification and the detection of associated complications, such as ILD or an underlying malignancy [2,8].

Cutaneous Manifestations

Cutaneous manifestations are the cornerstone of diagnosis in dermatomyositis and often the first sign of the disease. They are classified into pathognomonic, characteristic, and nonspecific lesions, and their pattern can point toward a particular serological subtype and prognosis [1].

Pathognomonic lesions include Gottron's papules, which present as erythematous-to-violaceous, flat-topped papules or plaques over the extensor surfaces of joints, primarily the knuckles, elbows, and knees, and the heliotrope sign, a violaceous, often edematous, erythema affecting the upper eyelids. These findings are highly specific for DM [1,2].

Among the characteristic lesions, poikiloderma in photo-exposed areas is a frequent finding, manifesting as the "V-sign" on the décolletage or the "shawl sign" on the back and shoulders. These areas exhibit a combination of epidermal atrophy, telangiectasias, and hyper- or hypopigmentation [1]. Another relevant manifestation is "mechanic's hands," characterized by hyperkeratosis, fissuring, and scaling on the lateral aspects of the fingers, which are more typical of antisynthetase syndrome but can be seen in DM [27].

Other lesions of great prognostic importance exist. Cutaneous ulcers, often painful and necrotic, can appear as a result of the underlying vasculopathy and are characteristically associated with the presence of anti-MDA5 antibodies [23]. On the other hand, calcinosis cutis, which involves the deposition of calcium salts in the skin and subcutaneous tissue, is more frequent in juvenile DM; however, in adults, it has been linked to anti-NXP2 antibodies [20]. Finally, microvascular involvement can be observed as periungual erythema and telangiectasias, reflecting the immune-mediated endothelial damage that defines the disease's pathophysiology [5,15].

Collectively, the cutaneous phenotype offers valuable clues: severe skin disease can be associated with anti-TIF1γ antibodies and a higher risk of cancer [17,18], while a florid and acute cutaneous onset may suggest the presence of anti-SAE antibodies [24]. Therefore, a thorough dermatological examination is fundamental not only for diagnosis but also as a "window" into the patient's immunological and systemic profile.

Muscular Manifestations

Muscle involvement is a hallmark feature of DM. The classic presentation includes symmetric and proximal muscle weakness with a subacute onset, progressing over several weeks to months [11,12,28]. Patients typically report difficulty performing tasks that require the use of the shoulder or pelvic girdles, such as getting up from a chair, climbing stairs, combing their hair, or lifting objects overhead. While myalgia may be present, especially in the initial phases, it is an inconsistent symptom and often mild compared to the magnitude of the weakness [1,28].

In addition to the limbs, involvement of the axial and pharyngeal musculature has significant prognostic relevance. Dysphagia, present in up to 58% of patients at some point in the disease, is due to weakness of the oropharyngeal and upper esophageal striated muscles and constitutes a risk factor for aspiration pneumonia and malnutrition [29]. Weakness of the neck flexor muscles can lead to "dropped head syndrome," while involvement of the paraspinal and abdominal muscles can make it challenging to sit up from a supine position and, in cases of severe weakness, lead to the development of camptocormia (an involuntary forward flexion of the trunk) [11].

To objectively quantify muscle damage, serum biomarkers are used, mainly muscle enzymes. Creatine kinase (CK) is the most sensitive marker, and its levels typically rise up to 10 times above the upper limit of normal in active phases [11]. Certain autoantibody profiles are associated with greater elevation; for example, patients with anti-Mi-2 antibodies frequently present with markedly elevated CK levels, consistent with their classic DM phenotype [21,30]. In contrast, it is crucial to note that CK levels do not always correlate with the severity of weakness; other patient subgroups may present with significant weakness despite having normal or only slightly elevated CK levels [3]. In these cases, other enzymes such as aldolase, lactate dehydrogenase, aspartate aminotransferase, and alanine aminotransferase can also be elevated and are of particular use [3,11].

Pulmonary Involvement and Other Systemic Manifestations

Beyond the skin-muscle axis, DM is a multisystem disease whose long-term prognosis largely depends on internal organ involvement. Pulmonary involvement is the most frequent and severe of these manifestations, although the heart, gastrointestinal tract, and joints can also be involved.

ILD is the leading cause of morbidity and mortality in patients with DM [12]. Its presentation is variable, ranging from asymptomatic radiological findings to progressive dyspnea with a dry cough. Certain autoantibody profiles are key predictors of the risk and phenotype of ILD. Antisynthetase antibodies (such as anti-Jo-1, PL-7, and PL-12) are strongly associated with a more chronic course of ILD [27]. In contrast, the presence of the anti-MDA5 antibody defines a high-risk subgroup for developing rapidly progressive ILD, a fulminant complication with high mortality, which can occur even in patients with little to no muscle weakness (amyopathic DM) [23]. Additionally, the co-expression of the anti-Ro52 antibody has been established as a marker of greater severity and poorer prognosis in patients with myositis-associated ILD [25,26].

Cardiac involvement, although often subclinical, can be potentially lethal. Manifestations include myocarditis, pericarditis, myocardial fibrosis leading to heart failure, and conduction system abnormalities that can generate arrhythmias or heart block [11,31]. Early detection via echocardiogram and cardiac magnetic resonance imaging is recommended in patients with symptoms or high-risk factors [31].

The most common gastrointestinal involvement is dysphagia due to weakness of the esophageal musculature, as previously mentioned. A rarer but serious complication, especially in DM associated with anti-NXP2, is vasculitis of the gastrointestinal tract, which can cause ischemia, ulcerations, and intestinal perforation [32]. Finally, articular manifestations are frequent, presenting as arthralgias or a non-erosive, symmetric arthritis that mainly affects the hands and wrists, and is a distinctive feature in patients with antisynthetase syndrome [12,27].

Clinical subtypes and immunological classification

DM is no longer considered a monolithic entity. Modern classification has evolved from a purely clinical description to an integrative approach that combines the patient's phenotype with their serological profile [12,28]. Using the information on MSAs previously described, it is possible to group patients into subtypes with distinct prognostic and therapeutic implications, which is fundamental in clinical practice [27].

Main Clinical Subtypes

The first layer of classification is based on the general clinical presentation. Classic DM is defined by the coexistence of proximal muscle weakness and characteristic cutaneous lesions [1,3]. In contrast, Amyopathic DM is diagnosed by the presence of typical cutaneous lesions for at least six months, but without clinically apparent muscle weakness [4]. This subgroup should not be considered benign, as it can be associated with severe systemic complications [4,23]. Finally, juvenile DM, although it shares features with the adult form, is distinguished by a higher incidence of vasculopathy and calcinosis, and an association with cancer that is extremely rare [12].

Dermatomyositis as a Paraneoplastic Syndrome and Serological Classification

In adults, DM is a recognized paraneoplastic syndrome, with a risk of malignancy that can reach 40% and that justifies systematic oncologic screening according to international guidelines [7,8]. The true power of modern classification lies in the use of MSAs to identify predictable phenotypes that inform not only about cancer risk, but also about the type of organ involvement, severity, and prognosis [12,27,28]. These clinico-serological correlations, which are the basis of current DM management, are summarized in Table 1.

**Table 1:** Clinico-serological Correlations of the Major Myositis-Specific Autoantibodies (MSAs) in Dermatomyositis ILD: Interstitial lung disease; DM: Dermatomyositis

Antibody	Clinical Phenotype and Main Features	Risk and Prognostic Associations	Typical CK Levels
Anti-Mi-2	Classic DM phenotype. Florid cutaneous manifestations (Gottron's, heliotrope) [26].	Good response to corticosteroid therapy. Low risk of malignancy and ILD [21,27].	Markedly elevated [30].
Anti-TIF1γ	Extensive and often severe, treatment-resistant skin disease [16,17].	The most robust marker for association with malignancy in adults [8,10,18].	Variable [16].
Anti-NXP2	Subcutaneous edema and severe myalgias [26].	Elevated risk of malignancy in adults. High incidence of calcinosis [20]. Can be associated with severe GI disease [32].	Variable [26].
Anti-MDA5	Predominantly amyopathic or hypomyopathic phenotype. Painful cutaneous ulcers, palmar papules, alopecia [23,27].	Very high risk of rapidly progressive ILD, with high mortality [23,25].	Normal or slightly elevated [23].
Anti-SAE	Acute onset with florid cutaneous disease, with mild or late-onset myositis [24].	Increased risk of malignancy [24].	Variable, often low at onset [24].
Antisintetasas (ej., Anti-Jo-1)	Defines "Antisynthetase Syndrome": myositis, ILD (chronic), non-erosive arthritis, "mechanic's hands," and Raynaud's phenomenon [12,27].	Prognosis is primarily marked by the severity of the ILD [12].	Moderately elevated [27].

This serological classification system has become an indispensable tool in clinical practice. The early identification of a patient's autoantibody profile enables the anticipation of the disease course, provides rational guidance in the search for complications, such as targeted oncologic screening or intensive pulmonary monitoring, and ultimately, leads to more personalized and precise therapeutic management [12,28].

Association with malignancy: risk and screening strategies

The association between DM and cancer is one of its most defining features, making this disease a potential paraneoplastic syndrome and its diagnosis a sentinel event for an occult malignancy [7,11]. Proper risk stratification and the implementation of an early detection protocol are fundamental to improving patient prognosis.

Overall Risk, Chronology, and Demographic Factors

Epidemiological studies and meta-analyses have shown that patients with DM have a 5- to 7-fold increased risk of developing cancer compared to the general population, with an absolute risk that can reach up to 40% in some series [7,12]. This risk is not constant over time; the chronology of tumor appearance is a key factor. The period of greatest danger is peri-diagnostic, spanning from the year prior to three years after the onset of DM symptoms, although the risk remains elevated for at least five years [8,10]. Certain demographic factors modify this risk. Age is the most important predictor, with a significant increase in risk in patients older than 40 years. Male sex has also been associated with a higher risk in some cohorts [7]. Furthermore, there is a geographic variation in the most frequent types of associated tumors, which often reflect the cancer epidemiology in each region (e.g., a higher frequency of ovarian cancer in European and North American cohorts, and nasopharyngeal cancer in Asian populations) [8].

Risk Stratification Based on Autoantibodies

The risk of malignancy is not uniform across all patients, and MSAs are the most powerful tool for its stratification, offering a level of precision that was unavailable when relying solely on older, less specific demographic and clinical risk factors. The presence of anti-TIF1γ antibodies confers the highest risk of all, with a probability of associated cancer that can exceed 70% in adults [10,18]. Likewise, anti-NXP2 antibodies are also considered a high-risk marker for neoplasia [8,20]. In stark contrast, patients with anti-Mi-2 or antisynthetase syndrome antibodies (such as anti-Jo-1) have a cancer risk that is not significantly higher than that of the general population [27,28].

Oncologic Screening Algorithms and Strategies

**Figure 1:**
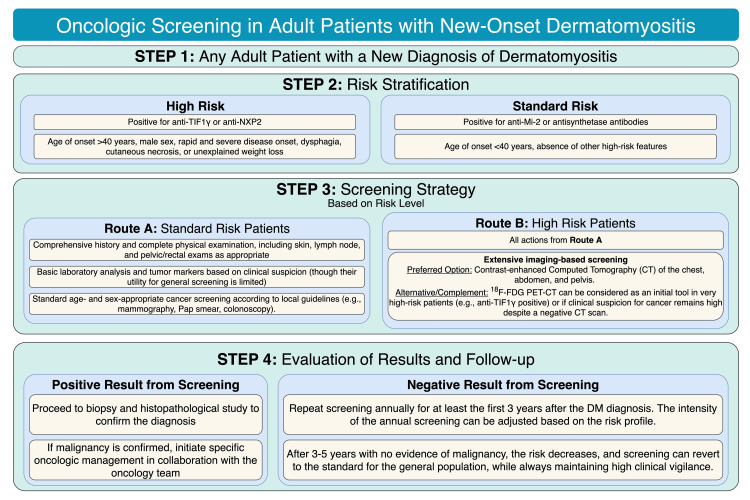
Proposed Algorithm for Oncologic Screening in Adult Patients with New-Onset Dermatomyositis DM: Dermatomyositis; MSA: Myositis-Specific Antibody; IMACS: International Myositis Assessment and Clinical Studies Group; CT: Computed Tomography; PET-CT: Positron Emission Tomography/Computed Tomography; TIF1γ: Transcription Intermediary Factor 1-gamma; NXP2: Nuclear Matrix Protein 2. Figure created by Dr. Gonzalez-Rodriguez using Draw.io.

Given the strong association, comprehensive oncologic screening is recommended for every adult patient with a recent diagnosis of DM, following a stepwise approach based on the risk profile, as proposed by the international guidelines from the IMACS (International Myositis Assessment and Clinical Studies Group) [8]. The initial evaluation for all patients includes a thorough medical history and physical examination, as well as basic laboratory analyses and standard age- and sex-appropriate cancer screenings (e.g., mammography, Pap smear, colonoscopy) [8,28]. For patients considered high-risk (advanced age, presence of anti-TIF1γ or anti-NXP2 antibodies, or alarm symptoms such as weight loss), a more in-depth workup is recommended. Contrast-enhanced computed tomography (CT) of the chest, abdomen, and pelvis is the most recommended imaging modality for this initial extensive evaluation [8]. Additionally, ¹⁸F-fluorodeoxyglucose positron emission tomography/computed tomography (¹⁸F-FDG PET-CT) has demonstrated high sensitivity in detecting occult neoplasms in this high-risk group. It is considered a valuable tool when clinical suspicion of malignancy is high and conventional studies are negative [33]. It is recommended to repeat the screening annually for at least the first three to five years after the diagnosis of DM, adjusting the intensity of follow-up according to the patient's individual risk profile [8]. Figure 1 summarizes the oncologic screening timeline for patients with DM.

Managing the oncologic risk in DM requires a personalized strategy that integrates demographic, clinical, and, critically, the patient's serological profile to guide rational and effective screening.

Comprehensive diagnosis

**Figure 2:**
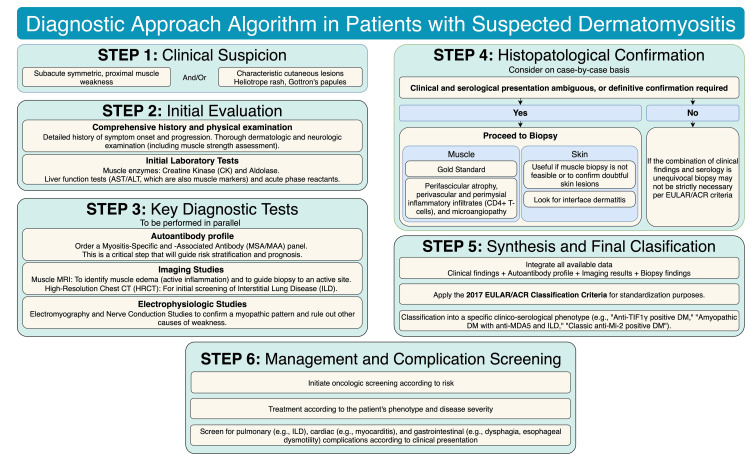
Diagnostic Approach Algorithm in Patients with Suspected Dermatomyositis DM: Dermatomyositis; CK: Creatine Kinase; ILD: Interstitial Lung Disease; MRI: Magnetic Resonance Imaging; HRCT: High-Resolution Computed Tomography; EULAR/ACR: European League Against Rheumatism/American College of Rheumatology. Figure created by Dr. Gonzalez-Rodriguez using Draw.io.

The diagnosis of DM is a complex process that requires a careful synthesis of the clinical presentation, laboratory findings, imaging studies, and histopathology. No single test is definitive, and the initial clinical suspicion must be corroborated through a multifaceted approach [3,12]. Figure 2 shows a diagnostic approach algorithm for patients with suspected dermatomyositis.

Classification Criteria and Laboratory Evaluation

To standardize the classification of inflammatory myopathies, the American College of Rheumatology and the European League Against Rheumatism (EULAR/ACR) developed criteria in 2017 based on a weighted scoring system [34]. It is essential to note that these are classification criteria for research, rather than strictly diagnostic criteria; however, they are of great utility in clinical practice. They assign points to variables across different domains: age of onset, muscle weakness (proximal, axial, dysphagia), characteristic cutaneous manifestations (heliotrope, Gottron's), the presence of the anti-Jo-1 antibody, muscle enzyme levels, and muscle biopsy findings. A cumulative score above a certain threshold classifies the patient as having "probable DM" or "definite DM," with or without a muscle biopsy [34]. In addition to muscle enzymes like CK and aldolase, acute-phase reactants such as the erythrocyte sedimentation rate and C-reactive protein may be elevated. However, they are nonspecific [3].

The Role of Imaging Studies

Imaging studies are crucial non-invasive tools for diagnosis and follow-up. Muscle magnetic resonance imaging (MRI) is the technique of choice for evaluating muscle involvement. It is highly sensitive for detecting muscle edema (hyperintensity on T2-weighted fat-suppressed or STIR sequences), which indicates active inflammation, and allows for the assessment of atrophy or fatty replacement as signs of chronic damage. Furthermore, MRI is fundamental for guiding a biopsy to an actively inflamed muscle, thereby increasing the diagnostic yield [12,35,36]. Nailfold capillaroscopy is a simple and non-invasive technique that allows for the visualization of the characteristic microangiopathy of DM, showing dilated capillary loops, capillary dropout, and microhemorrhages [37]. Finally, as previously mentioned, PET-CT can play a dual role: searching for an occult malignancy and evaluating the extent of systemic inflammation, including myositis, vasculitis, and lung disease [33].

Histopathological Findings on Biopsy

Biopsy remains the "gold standard" for diagnostic confirmation in doubtful cases. A muscle biopsy from a clinically weak or MRI-active muscle reveals characteristic findings: perifascicular atrophy is the most specific finding for DM. Additionally, an inflammatory infiltrate composed mainly of CD4+ T-cells, B-cells, and plasmacytoid dendritic cells is observed, located predominantly in the perivascular and perimysial areas, unlike in polymyositis, where the infiltrate is endomysial [11,15]. Deposits of the membrane attack complex (C5b-9) can also be detected in endomysial capillaries, providing evidence of the immune-mediated microangiopathy [13]. A skin biopsy from an active lesion is also very useful, showing an interface dermatitis with vacuolar degeneration of the basal layer of the epidermis, a perivascular lymphocytic infiltrate, and, often, increased mucin deposition in the dermis [1].

Autoantibody Profile as a Diagnostic Tool

The analysis of the MSA and MAA profile has become a pillar of modern diagnosis [26,28]. As detailed in the classification section, the identification of an MSA not only strongly supports the diagnosis but also predicts the clinical phenotype and risk of complications. For example, the detection of anti-Mi-2 antibodies in a patient with compatible symptoms is highly specific for classic DM [21,22]. Positivity for anti-Jo-1 points toward antisynthetase syndrome, while positivity for anti-TIF1γ mandates a comprehensive search for cancer [8,10]. Therefore, autoantibody testing is no longer considered a mere supplement but rather an essential and integral part of the diagnostic process that should be performed in every patient with suspected DM [12,27].

Current treatment and management of complications

The treatment of DM aims to achieve multiple objectives: controlling inflammatory activity to improve muscle strength and resolve cutaneous manifestations, managing systemic complications such as ILD, minimizing drug toxicity, and improving the patient's quality of life [11,12]. The therapeutic approach must be individualized, based on disease severity, the organs affected, and, increasingly, the patient's autoantibody profile [26,27].

First-Line Treatment

Systemic glucocorticoids are the cornerstone for inducing remission in DM, especially in patients with significant muscle involvement [3,38]. Treatment is usually initiated with high doses of prednisone (0.5-1 mg/kg/day), followed by a gradual and progressive dose reduction once disease control is achieved, to mitigate its well-known adverse effects [11,38]. To facilitate this tapering and for long-term maintenance of remission, a "corticosteroid-sparing" immunosuppressive agent is introduced early. The most commonly used drugs in this context are azathioprine and methotrexate [38]. Mycophenolate mofetil is another first-line option, considered especially useful in patients with coexisting cutaneous or pulmonary involvement [1,38].

Therapies for Refractory or Severe Disease

For patients who do not respond adequately to first-line therapy or who present with severe, life-threatening manifestations, second-line options exist. Intravenous immunoglobulin is an effective treatment for refractory DM, proving particularly useful in cases of severe dysphagia, resistant skin disease, and for achieving rapid control of muscle activity [11,38]. On the other hand, rituximab, a monoclonal antibody that depletes CD20+ B cells, has been established as a valuable option for refractory cases involving the muscular, cutaneous, and pulmonary systems, based on the pathogenic role of B lymphocytes in DM [38].

New Targeted Therapies and Emerging Approaches

The evolution in our understanding of DM's pathophysiology has allowed the therapeutic landscape to move beyond broad, non-specific immunosuppression toward targeted therapies. Janus kinase (JAK) inhibitors, such as tofacitinib or baricitinib, represent one of the most significant therapeutic breakthroughs of the last decade [39]. By blocking the type I interferon signaling pathway, which is central to the pathogenesis of DM, they have shown remarkable efficacy in treating refractory cutaneous disease and, very relevantly, in managing ILD, including the rapidly progressive forms associated with the anti-MDA5 antibody [38,39]. Other biologic therapies, such as abatacept, have also been explored in selected cases, although with more limited evidence [38].

Specific Management of Complications

The treatment of systemic complications often requires a particular approach. The management of ILD is challenging, and its strategy depends on the severity and speed of progression. In addition to corticosteroids, immunosuppressants such as mycophenolate mofetil or cyclophosphamide (the latter used in more severe or progressive cases) are employed, as are, increasingly, therapies like rituximab or JAK inhibitors [12,38,39]. Calcinosis is notoriously difficult to treat, and there is no standard therapy, although partial success has been reported with various drugs such as bisphosphonates, diltiazem, and rituximab, among others [12]. Finally, cutaneous manifestations, in addition to systemic treatment, benefit from topical therapies such as corticosteroids and calcineurin inhibitors. Antimalarials like hydroxychloroquine can be effective but must be used with caution due to the risk of exacerbating photosensitivity [1,12].

Prognosis and follow-up

The prognosis of DM has improved markedly in recent decades thanks to earlier diagnosis and more effective therapies; however, it remains a chronic disease with the potential for significant morbidity and mortality [11,12]. The course of the disease is highly variable and is strongly influenced by the patient's age, the presence of severe systemic complications, and, critically, by the autoantibody profile [12,28].

Clinical and Serological Prognostic Factors

Several factors help predict the evolution of the disease. Indicators of a poorer prognosis include advanced age at diagnosis [7], the presence of an underlying malignancy [8], a delay in treatment initiation [38], cardiac involvement [31], and severe muscle weakness, especially if accompanied by dysphagia due to the risk of aspiration pneumonia [11,29]. ILD is one of the most important predictors of mortality [12]. The serological profile is equally fundamental for prognostic stratification. Antibodies such as anti-TIF1γ and anti-NXP2 are associated with a poorer prognosis due to their strong link to cancer [8,10,18,20]. The presence of anti-MDA5 is a marker of high mortality due to its association with rapidly progressive ILD, while the coexistence of anti-Ro52 can worsen the prognosis of any myositis-associated ILD [23,25]. In contrast, patients with anti-Mi-2 antibodies generally have a more favorable prognosis, with a good response to treatment and a low risk of severe systemic complications [21,27].

Monitoring Disease Activity and Relapses

Follow-up of patients with DM must be continuous and systematic to detect relapses and adjust treatment. Monitoring includes regular clinical assessments of muscle strength, often using standardized tools such as the Manual Muscle Testing 8 (MMT-8), as well as evaluation of cutaneous disease activity [38]. It is essential to actively inquire about symptoms suggesting other organ involvement, such as dyspnea, cough, or difficulty swallowing. Muscle enzymes such as CK are useful for monitoring, but it is important to remember that their levels may not directly correlate with disease activity, especially in patients undergoing treatment or with certain serological subtypes [28,30]. Furthermore, patient-reported outcomes and quality of life, which are key aspects of comprehensive follow-up, should be assessed [38].

Recommendations for Multidisciplinary Follow-up

Given the multisystemic nature of DM, optimal patient management requires a coordinated approach by a multidisciplinary team [12,38]. The rheumatologist typically serves as the primary coordinator of immunosuppressive treatment. Close collaboration with a dermatologist is essential for managing cutaneous manifestations, which can be very resistant. The pulmonologist plays a key role in the diagnosis and follow-up of ILD. A specialist in physical medicine and rehabilitation, along with physical and occupational therapists, is fundamental for designing exercise programs to help recover muscle strength and functionality. Depending on the complications, a cardiologist, a gastroenterologist, and, in the case of malignancy, an oncologist may also be necessary. This comprehensive approach is the most effective strategy for optimizing long-term outcomes in this complex disease [12].

## Conclusions

DM is a complex autoimmune disease whose understanding has evolved significantly, from being viewed as a simple inflammation of the skin and muscle to being recognized as an immune-mediated systemic microangiopathy, with a pathophysiology dominated by the type I interferon pathway. The recognition of its heterogeneous clinical manifestations, which range from classic cutaneous lesions to pulmonary and cardiac involvement, is fundamental for its diagnosis. The modern era in the management of dermatomyositis is characterized by the central role of specific autoantibodies, which have evolved from being mere diagnostic markers to indispensable tools for risk stratification. The identification of high-risk profiles, such as the association of anti-TIF1γ with malignancy or anti-MDA5 with rapidly progressive lung disease, allows for personalized medicine. Consequently, the prognosis and quality of life for patients critically depend on an early diagnosis and a proactive, multidisciplinary approach, where surveillance for complications is guided by the individual's clinical and serological profile.
